# The effects of a dual task on gaze behavior examined during a simulated flight in low-time pilots

**DOI:** 10.3389/fpsyg.2024.1439401

**Published:** 2024-11-19

**Authors:** Naila Ayala, Suzanne Kearns, Elizabeth Irving, Shi Cao, Ewa Niechwiej-Szwedo

**Affiliations:** ^1^Department of Kinesiology and Health Sciences, University of Waterloo, Waterloo, ON, Canada; ^2^Waterloo Institute for Sustainable Aeronautics, University of Waterloo, Waterloo, ON, Canada; ^3^Department of Geography and Aviation, University of Waterloo, Waterloo, ON, Canada; ^4^School of Optometry and Vision Science, University of Waterloo, Waterloo, ON, Canada; ^5^Department of Systems Design Engineering, University of Waterloo, Waterloo, ON, Canada

**Keywords:** gaze behavior, cognitive load, dual task, auditory oddball paradigm, eye movements and visual attention, aviation

## Abstract

Cognitive load can impair an operator’s ability to optimally scan and process relevant information that is critical to the safe and successful operation of an aircraft. Since the cognitive demands experienced by pilots fluctuate throughout a given flight due to changes in task demands that range from high to low cognitive load, it has become increasingly important to objectively track and quantify these changes accordingly. The analysis of eye movements has been shown to be a promising method to understand information acquisition, processing efficiency, and how these aspects of cognition impact pilot performance. Therefore, the aim of the current study was to assess the impact of a dual task paradigm on low-time pilot flight performance and gaze behavior during two phases of flight with varying levels of cognitive load. Twenty-two licensed pilots (<350 h) completed simulated flight circuits alongside an auditory oddball task under visual flight rules conditions. Self-reported situation awareness scores and auditory task performance revealed the dual task was more demanding than the single tasks. Flight performance and gaze behavior indicated that primary task performance and information processing remained unaffected. These results suggest that the recruited pilots attained a level of skill proficiency that enabled the efficient deployment of cognitive resources to successfully complete the flying task under states of increased cognitive load. Combined with previous research findings, the results suggest that the effect of secondary tasks depends on the type of tasks used (i.e., simple/choice response tasks, memory recall, etc.). The utility of using a dual task and gaze behavior to probe flight proficiency and information processing efficiency throughout training are discussed.

## Introduction

Today’s intelligent aircraft cockpit provides multisource information via an array of instrument panels and controls that are associated with different procedures that a pilot must attend to at any given moment (i.e., pilot monitoring) (Federal Aviation Administration, 2021). The capacity limitations associated with human information processing make the task of acquiring information from these various areas of interest (AOIs) challenging, particularly during states of high cognitive load (i.e., high amounts of cognitive resources demanded from the operator by competing activities) (Engström et al., 2017). Therefore, it is of no surprise that ineffective pilot monitoring was cited as being a major contributor to human factor errors during cognitively demanding phases of flight (i.e., take-off, approach and landing) (Boeing, 2021; National Transportation Safety Board, 1994). Evidently, a pilot’s ability to select and monitor relevant information in such a dynamic environment are critical to flight success and safety. Therefore, being able to objectively track and quantify changes in information processing under varying cognitive load conditions is an important line of inquiry as it provides a framework to understand how operators effectively monitor task-critical sources of information necessary for successful task performance during states of high cognitive load.

Since vision plays a critical role in how we interact with our environment, eye tracking serves as a non-invasive method that reveals discrete cognitive processes and strategies used to facilitate skill performance that are not readily observable through overt behaviors alone (Ayala et al., 2022; Vickers and Williams, 2017). Indeed, where we look (i.e., fixate) is governed by ongoing perceptual (i.e., bottom-up, lower-level stimulus futures) and cognitive (i.e., top-down, higher-level mental hierarchies and goals) processes that facilitate the selection and processing of relevant information that support skill performance (Engström et al., 2017; Hudspeth et al., 2013; Land and Hayhoe, 2001). For instance, gaze (i.e., coordinated head-eye movements) patterns have been suggested to be predominantly driven by top-down information hierarchies (i.e., scripts) rather than the ‘intrinsic salience’ of objects, particular in populations that have experience with the task being performed (Land and Hayhoe, 2001). Traditional approaches to analyzing gaze behavior have supported the claim that top-down priorities guide visual scanning based on discrete measures of total proportion of fixation time (i.e., dwell time), fixation frequency or average fixation durations toward specific areas of interest (AOIs). Specifically, these traditional metrics demonstrate what sources of information are crucial to the task at hand based on where gaze is allocated for larger proportions of total dwell time as well as higher fixation frequencies (Brams et al., 2018; Land and Hayhoe, 2001). Average fixation duration can also serve as an indicator of information processing efficiency, where longer durations are associated with more time being required to process the information being fixated and shorter durations are associated with less time processing objects of interest (i.e., typically associated with quickly verifying information or highly efficient scanning) (Brams et al., 2018; Land and Hayhoe, 2001). Other computationally complex measures of gaze behavior have also been applied to identify global patterns of behavior that emerge over the course of task performance such as stationary gaze entropy (SGE), gaze transition entropy (GTE), and cognitive tunneling (i.e., the frequency and duration of poor internal cockpit monitoring events) (Ayala et al., 2024). SGE and GTE have emerged more recently in the literature to provide an objective indices of gaze dispersion and fixation sequence complexity, respectively (Shiferaw et al., 2019). While SGE values are modulated by the extent of fixation dispersion (i.e., low entropy = concentrated fixation distribution across very few AOIs; high entropy = dispersed fixation distribution across many AOIs), GTE is more reflective of visual attention deployment. An exploratory (i.e., salience-based) mode of attentional deployment is associated with higher GTE (i.e., less attentional bias in fixating several areas that are relevant and/or irrelevant to the task at hand), while a reduction in GTE is reflective of a focal mode of visual attention (i.e., structured, and biased attention toward fewer AOIs), which may be more indicative of task-engagement (Shiferaw et al., 2019). In contrast, cognitive tunneling is a dynamic gaze metric that identifies when individuals’ fall into a gaze pattern of hyper fixation toward a singular AOI for an extended period of time. The examination of hyper fixation is of interest because it represents a lack of visual scanning to other sources of information in the environment that may hold pertinent information crucial to pilot SA and successful task completion (Ayala et al., 2023; Ayala et al., 2024). Although more research is required to fully understand how changes in cognitive load influence gaze entropy and cognitive tunneling measures, these metrics have been shown to be useful in providing additional information in parallel with traditional gaze metrics and behavioral performance about the spatiotemporal properties of gaze behaviors associated with information processing and goal-directed actions (Ayala et al., 2022).

Previous research has examined the question of cognitive load primarily by employing a dual task paradigm wherein the difficulty of performing a primary task (i.e., flying) is made more challenging by the addition of a simultaneous secondary task (i.e., visual search, mental arithmetic, auditory tone, etc.); thereby, introducing additional rules and goals to be maintained/manipulated in working memory (Engström et al., 2017). As a result of the limited human brain capacity, increased cognitive load associated with multitasking has often resulted in impaired behavioral performance (Engström et al., 2017). Indeed, numerous studies involving machine operated simulation tasks (i.e., driving, flying) have used dual-task paradigms to shown that operator control, problem solving, decision making and, most importantly, gaze behavior are significantly impacted when the demand for cognitive resources is high (Allsop and Gray, 2014; Engström et al., 2017; Kanaan and Moacdieh, 2022; Van de Merwe et al., 2012; Yang et al., 2018). Specifically, high cognitive load has been shown to be associated with a narrowing of attention toward fewer areas of interest (AOIs), reduced SGE, more frequent fixations and longer fixation durations (Allsop and Gray, 2014; Di Nocera et al., 2007; Engström et al., 2017; Kanaan and Moacdieh, 2022; Lu et al., 2020; Lutnyk et al., 2023; Reimer, 2009; Wright et al., 2014). These changes in gaze behavior were reported in parallel with increased misjudgment (i.e., spatial disorientation, increased time and error rates associated with hazard object/event detection), reduced aircraft control (i.e., increased variability in aircraft speed, pitch and turn coordinator), and unstable approaches and landings (Crosby and Parkinson, 1979; Engström et al., 2017; Ziv, 2016). Collectively, these findings suggest that increases in cognitive load due to multitasking are associated with increased processing time toward fewer sources of information because of increasing demands placed on cognitive resources, which increases the risk of human error and unsafe machine operation.

A more extensive review of dual-task paradigm literature revealed that the effects of cognitive load on complex tasks like driving a car are strongly selective and task dependent (Engström et al., 2017). For instance, several driving studies demonstrated no evidence for increased crash/near-crash risk associated with primarily cognitive tasks, such as talking on the phone or using a radio (Simmons et al., 2016). In accounting for these apparent inconsistent and counterintuitive findings regarding the effects of cognitive load on driving performance, the Cognitive Control Hypothesis was proposed. The hypothesis states that cognitive load will selectively impair performance on tasks that require *controlled* processing (i.e., novel, uncertain, nonroutine tasks that require top-down executive functions like working memory), while leaving tasks that require *efficient* processing (i.e., practiced, effortless, generally unconscious tasks that require little to no cognitive control oversight) (also known as *automatic* processing) relatively unchanged (Engström et al., 2017). Notably, the transition of a learned skill from controlled processing to efficient processing represents a progression from low to high efficiency in the cognitive processes needed to plan, execute, and update skilled movements (Immink et al., 2020). Since the majority of pilot monitoring errors occur during cognitively demanding phases of fight (i.e., take-off, approach and landing) (Boeing, 2021; National Transportation Safety Board, 1994), the Cognitive Control Hypothesis suggests that these phases of flight are associated with controlled performance (i.e., require cognitive resources to manage the respective sub-tasks, while maintaining aircraft control) and are susceptible to increases in cognitive load, more so than any other flight phase (i.e., cruise). Accordingly, a dual-task paradigm could serve as a useful method to understand how changes in cognitive load impact information processing via changes in gaze behavior across the various phases of flight.

This paper aimed to build on previous aviation-related work employing a dual task paradigm to examine the utility of gaze metrics in assessing the impact of cognitive load on pilot’s performance and information processing using a more ecological approach. There are two major aspects through which this study differs from previous work. First, the current work sought to use a dual task paradigm to objectively probe the impact of a secondary task on cognitive load across two phases of a flight circuit (i.e., cruise [low cognitive load], approach and landing [high cognitive load]). Since it has been well established that the two stages of flight (cruise, approach and landing) vary in the extent to which they require cognitive resources to monitor and complete the required sub-tasks (Di Nocera et al., 2007; Federal Aviation Administration, 2021; Wilson, 2002), it is important to examine how the imposed change in cognitive load impacts the phase-specific spatiotemporal aspects of gaze behavior. Crucially, accurately monitoring pilot’s cognitive load through continuous, objective, gaze-based metrics is of great significance for the development of real-time metrics to assess pilot’s information processing capability and may also be an effective method to track pilot’s progression in the development of efficient information processing throughout training. Second, the current work was designed to have high fidelity to increase the generalizability of the findings to low-time pilots. Specifically, the dual task involved monitoring auditory inputs to simulate the modality of Air Traffic Control [ATC] calls, while simulating the flight environment with a fully immersive flight simulator. This is distinct from previous work that used video recordings for the primary task (van de Merwe et al., 2012), introduced secondary tasks that did not resemble general aviation-like procedures (i.e., mental arithmetic, working memory tasks, visual search, military specific shooting tasks, etc.) (Abel, 2009; Allsop et al., 2017; Babu et al., 2019), and recruited participants with no aviation experience (Allsop and Gray, 2014; Lutnyk et al., 2023).

The current study used a secondary task as a probe to evaluate the impact of cognitive load on flight performance and gaze behavior across two phases of flight: cruise and landing. It was hypothesized that performance on the auditory oddball will be significantly less accurate and slower during the approach and landing phase of flight compared to the cruise phase. Moreover, based on previous work using a VFR (Visual Flight Rules) paradigm, it was hypothesized that increased cognitive load will be associated with a reduction in attention allocation across a smaller set of task-relevant AOIs manifested as an increase in dwell time and fixation durations toward the external environment (i.e., front window) (Allsop and Gray, 2014; Allsop et al., 2017; Kanaan and Moacdieh, 2022; Yang et al., 2018; Yang et al., 2022). Although more dynamic measures of gaze behavior have only been examined in the context of increasing visuomotor task difficulty (i.e., wind manipulations) (Ayala et al., 2023), it was expected that an increase in cognitive load would similarly be associated with a reduction in the dispersion of fixations (SGE), a reduction in visual scan pattern complexity (GTE), and an increase in cognitive tunneling. These gaze behavior changes are expected to be specifically evident during the approach and landing rather than the cruise phase due to the increased cognitive load induced by processing task-specific information to ensure safe landing, which may overlap with the processing demands of the secondary auditory task.

## Methods

### Participants

Twenty-two participants (14 males, 8 females; age range 18–24 years, mean = 21 years old, SD = 2) were recruited from the student populations at the University of Waterloo. All participants were current aviation students or individuals who had obtained at least their private pilot’s license (PPL) (number of flight hours range: 48–340 h, mean = 199 h, SD = 73; PPL = 16; CPL = 6). All participants had normal or corrected-to-normal vision and had not been previously diagnosed with a neuropsychiatric/neurological disorder or learning disability. Participation was voluntary, and participants received $25/h as remuneration. The University of Waterloo Research Ethics Board Committee (#43564) approved the study protocol, which was performed in accordance with the 2008 Declaration of Helsinki. Consent was obtained prior to beginning the protocol. Note that one participant was excluded from analysis as a result of corrupt data files.

### Experimental setup and apparatus

#### Flight simulator

**Figure 1:**
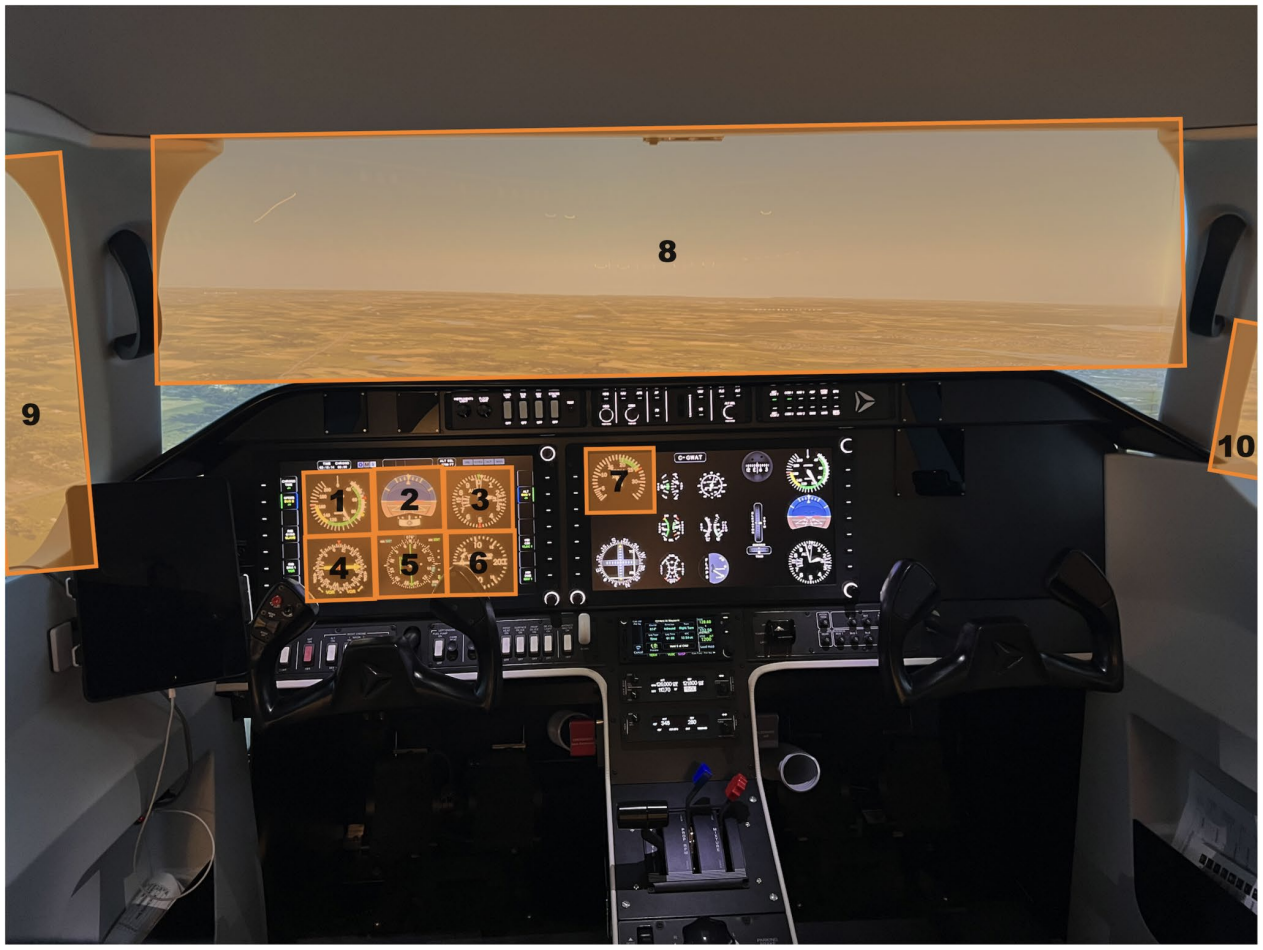
Illustration of the visual stimuli employed in the AL250 flight simulator environment. The participants point of view of the cockpit replicated that of a pilot flying a Cessna 172, pre-set for a cruise and landing approach to Waterloo International Airport, Breslau, Ontario, CA. The orange boxes represent the ten main areas of interest used in the gaze analyses. These include the airspeed (1), attitude (2), altimeter (3), turn coordinator (4), heading (5), vertical speed (6) and power (7) indicators, as well as the front (8), left (9), and right windows (10).

An AL250 ALSIM flight simulator (ALSIM, France) configured as a generic single engine aircraft that is representative of a Cessna 172 was used with the necessary instrument panel (steam gauge configuration), an avionics/GPS system, an audio/lights panel, a breaker panel, and a Flight Control Unit (FCU) (see Figure 1). The field of view covered by the simulator was 250° by 49° via panoramic VFR-VR-HD projectors. The participants sat in a height-adjustable seat and controlled the aircraft with a yoke, throttle lever, and rudder pedals. Stimuli presentation, and behavioral data collection and acquisition were controlled from the Instructor Station and Engineering pack (ALSIM, France).

#### Eye tracker

MindLink eye-tracking glasses (AdHawk Microsystems Inc., Waterloo, ON, Canada) were used to track the participants’ gaze (i.e., eye and head) movements. MindLink is a non-camera-based eye tracker embedded in a frame of eyeglasses that uses an ultra-compact micro-electromechanical system (MEMS) to track the eye and gaze movements (Zafar et al., 2023). The eye tracker was operating at 250 Hz, transmitting the gaze data and the video of its front-facing camera (82° field of view, 1080p, 30 Hz) via the AdHawk eye tracking software to a laptop (60 Hz refresh rate, 1920 × 1,080 pixels, Microsoft 11) visible only to the experimenter.

#### Scenario and task

Participants were tested in a single experimental session (~90 min) which started with a visual screening that included a visual acuity test (Bailey-Lovie chart) and a stereoacuity test (Randot Stereo test, Stereo Optical Company Inc.). A pilot briefing (led by an instructor pilot) and practice trial were completed to familiarize the participants with the simulator environment (Al 250, ALSIM, France), flight path, and flight parameters (cruise airspeed [110 kts] and altitude [2017 ft], landing approach airspeed [65 kts] and touchdown reference point [center of 500-foot marker]). The experimental scenarios were programmed in the flight simulator environment, flying into the Region of Waterloo International Airport (CYKF; Runway 26), Breslau, Ontario, Canada. Participants were then asked to complete a total of 9 customized trials while their gaze movements were recorded. The trials were pseudo-randomized (i.e., ABC, BCA, CBA, BAC) into three types of scenarios: (1) single-task auditory oddball task (control condition), (2) single-task flying circuit (cruise [low cognitive load] and landing [high cognitive load]), and (3) dual task paradigm (single task circuit with auditory oddball task). Prior to beginning the experimental trials, a 9-point eye-tracking calibration and validation procedures were completed by the examiner (average gaze error < 2°).

**Figure 2:**
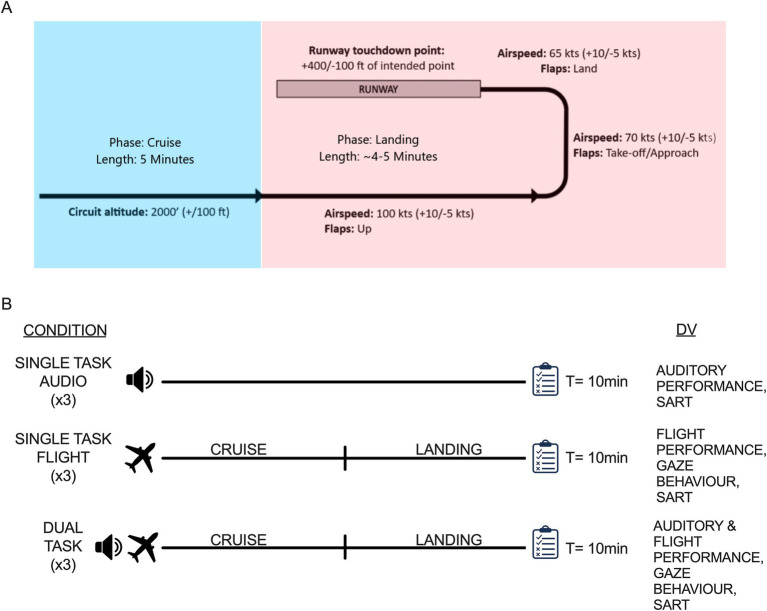
Illustration of the simulated flight path and circuit parameters for cruise (easy [blue]) and landing (difficult[pink]) phases of flight (A). Summary of the task conditions, scenario design, and the dependent variables assessed for each type of trial (B). Three trials of each condition were completed for a total of 9 trials, which were pseudorandomized. Single task audio trials assessed response time and accuracy in distinguishing target (30%) tones among the more commonly presented non-target (70%) tones. The exact timing of auditory stimuli presentation varied across and within trials with an inter-stimulus interval range of 1900 msec to 2,100 msec. The single task flight trials assessed flight performance and gaze behavior as participants flew the cruise and landing scenario. The dual task condition involved participants completing the flight and auditory tasks simultaneously. The dual task trials assessed auditory and flight performance as well as gaze behavior. Each trial lasted approximately 10 min for a total session duration of 90 min. Each trial was also followed by the Situational Awareness Rating Technique (SART) questionnaire (i.e., clipboard represents the online questionnaire participants completed).

For the flight task, all participants received the exact same environmental configurations in Visual Flight Rules (VFR) conditions (i.e., high visibility [>20 miles] and calm [0 kts, 0°]) with a pre-set start at the beginning of the cruise stage of the flight circuit to the airport at an altitude of 2017 ft., with flaps and trim set to zero, and a starting speed and RPM of 110 kts and 2000 rpm, respectively. The flying task was segmented into two cognitive load phases. Cruise was the first phase of flight wherein the goal was to maintain aircraft control (i.e., aircraft altitude [2017 ft], airspeed [100–110 kts], and vertical speed [0 fpm]) by monitoring the gauges for any slight deviations in speed or altitude and adjusting the flight controls as necessary. The second phase consisted of landing the plane wherein the goal was to land the plane as smoothly and accurately as possible relative to the center of the 500 ft. markers near the start of runway 26. Note that this phase consists of several aircraft configuration changes that required specific, time-sensitive maneuvers that allowed the plane to descend and become aligned with the runway in a safe and stable manner. The trial was terminated after the participant brought the plane to a complete stop, or if the landing was deemed unsuccessful (i.e., plane crash or plane landed off the runway). Note that pilots were required to communicate with Air Traffic Control (ATC) during both phases of flight, as they normally would in a real aircraft. Figure 2 illustrates the experimental overview including the three task conditions, and the simulated scenario along with the flight path and circuit parameters.

Auditory oddball task (control task) used auditory tones that were created using VPixx 3.2.1 software and were presented biaurally at 80 decibels using Apple computer speakers. Participants remained in the left pilot seat of the cockpit with the aircraft parked on the runway. Before testing, participants were presented with an iteration of high (1,000 Hz) and low (375 Hz) tones and asked if they could discriminate between the two tones. Participants were then instructed to respond by pressing on the pilot push-to-talk button located on the yoke with their left hand as quickly as possible when hearing a high “target” tone (probability = 30%), and to withhold a response when hearing a low “non-target” tone (probability = 70%). Participants were notified when a trial began by the instructor followed by an iteration of high and low tones randomly presented at inter-stimulus intervals ranging from 1900 msec to 2,100 msec.

The dual task paradigm condition involved performing both the flying circuit and auditory oddball tasks simultaneously. Participants were informed by the instructor when the trial began. The auditory tones were presented and responded to just as they were during the auditory only condition. Every participant was given the same instructions to “primarily focus on flying the aircraft in the dual task condition; therefore, if the task becomes too demanding you should focus on flying the aircraft more than attending to the auditory task.”

After each experimental trial (i.e., single flying circuit, single auditory oddball task, dual task paradigm) the participant was then asked to complete the Situational Awareness Rating Technique (SART) questionnaire to gauge their subjective opinion on various domains related to task difficulty, and the supply and demand of attentional resources required during task performance (Taylor and Selcon, 1990).

### Data processing and analysis

Based on the various flight performance measures collected, analysis was split across the two phases of flight: (1) Cruise performance- includes airspeed and vertical speed mean and variability- and (2) Landing performance- includes completion time (sec; duration of time from the start of the flying scenario to the plane coming to a complete stop on the runway), landing accuracy (degrees; the difference between the center of the plane and the center of the 500 ft. runway marker at point of touchdown). Notably, the distinct cruise and landing performance metrics were not continuous across phases. For instance, vertical speed would only be an appropriate measure for landing performance during the final phase of landing but not necessarily all other parts of the landing phase (i.e., downwind, base). Additionally, the required mean airspeed was expected to constantly decrease throughout the landing phase whereas during cruise, there is an expected consistent mean airspeed that participants are expected to maintain. All flight metrics were examined within their respective phase of flight and subjected to a repeated measures ANOVA with a single within-subject factor (task condition: single, dual). Participants were instructed to prioritize the primary flight task when the secondary task became too challenging to manage. As such, it was expected that flight performance would remain similar across the single flight task and dual task conditions for both phases of flight (i.e., cruise, landing).

Auditory oddball task performance was assessed using auditory response time (msec; response time to accurate target tones) and accuracy (percent; percentage of correct responses to total number of tones presented). Auditory oddball performance was examined during single auditory task conditions, as well as the dual task condition. Since the dual task condition was split into a cruise (low load) and landing (high load) phase of flight, raw auditory response data were subjected to a repeated measures ANOVA (within-group factor: cognitive load [single auditory task, dual cruise, dual landing]). To specifically examine the impact the dual task had on auditory responses during the different flight phases, the dual task ‘cost’ (DTC) (Equation 1) for auditory response metrics were calculated by expressing participants’ changes in the respective measures during dual task performance relative to their control task (i.e., auditory only) performance according to the formula (Zhou et al., 2014; Manor et al., 2010; Schwenk et al., 2010):


(1) $$DTC=\left[\frac{\left(\mathrm{dual}\;\mathrm{task}\;\mathrm{performance}-\mathrm{control}\;\mathrm{task}\;\mathrm{performance}\right)}{\mathrm{control}\;\mathrm{task}\;\mathrm{performance}}\right]\times 100$$

Dual task cost comparisons for auditory response were conducted with a repeated measures ANOVA (within-group factor: phase of flight [cruise, landing]). Previous work has shown that DTC increases with increasing cognitive load. Therefore, DTC for response time and accuracy is expected to increase significantly more in the landing phase of flight compared to the cruise phase of flight.

Gaze data were post-processed offline using a custom-made script that used the 3D gaze vectors provided by AdHawk software for saccade and fixation detection, similar to Ayala et al. (2024). Eye-movement traces were visualized by the experimenter and played back at a slowed speed superimposed over the video displaying the simulator environment. The task environment was discretized using a custom code that partitioned the simulator environment into ten areas of interest (AOIs) (Figure 1). The AOIs were manually defined to represent airspeed (1), attitude (2), altimeter (3), turn coordinator (4), heading (5), vertical speed (6), and power (7) cockpit indicators, along with the front (8), left (9), and right (10) windows. In line with previous work, fixations found outside these AOIs were defined as non-areas of interest and were excluded from analysis (<5%). Trials with missing data (i.e., loss of signal >30% found in <8% of trials) and outliers from each of the dependent variables (i.e., >1.5 the interquartile range around the first and third quartile found in <1% of trials) were removed.

Gaze behavior was examined across each phase of flight (cruise [low load], landing [high load]) using traditional and advanced (i.e., static and dynamic) gaze measures (Ayala et al., 2022; Ayala et al., 2023; Ayala et al., 2024; Glaholt, 2014). Traditional gaze-based analysis included the calculation of dwell time (%; total dwell duration spent within a given AOI as a function of total flight time) and average dwell duration (msec; average duration of uninterrupted dwells within a given AOI). Static entropy-based analyses were completed using the ten AOIs outlined in Figure 1 (Ayala et al., 2023). Custom scripts (Ayala et al., 2023) were used to compute both SGE (Equation 2; Shannon, 1984) and GTE (Equation 3; Ciuperca and Girardin, 2007), which were then normalized (Equation 4; Shannon, 1984).

Specifically, SGE was computed by first producing a vector, V, of length 10 (for each AOI), where V_i_ was the total number of fixations at AOI i. V was then divided by the total number of fixations in the sequence, so that V_i_ was the probability of a fixation landing at AOI i. The probability vector V was then applied to Equation 2.


(2) $$H_{SGE}\left(V\right)=-\underset{v\in V}{\sum }v\cdot \log\left(v\right)$$

GTE was computed by first creating a 10×10 (AOI) transition matrix, M, where M_i,j_ was the total number of transitions from AOI i to AOI j. Each row, M_i,∗_, was divided by the sum of row i, so that M_i,∗_ represented the probability of fixation transition from AOI i to any of the ten AOIs. Finally, GTE was computed using Equation 3 (Ciuperca and Girardin, 2007), applying the transition matrix M and the probability vector V.


(3) $$H_{GTE}\left(M\right)=-\overset{10}{\underset{i=1}{\sum }}V_{i}\overset{10}{\underset{j=1}{\sum }}M_{i,j}\cdot \log\left(M_{i,j}\right)$$

(4) $$H_{\mathrm{NORMAL}}=H/H_{\mathrm{MAX}}$$

The dynamic entropy-based analysis was completed using the 10 s average sliding window developed by Ayala et al. (2023). This cognitive tunneling analysis was used to examine the reduction in gaze transitions from the external environment to the internal cockpit environment. This analysis specifically quantified the number of cognitive tunneling bouts (i.e., number of instances in which a dwell remained entirely outside of the cockpit for at least 10 s), bout duration (sec; the average duration of all bouts that occurred in a trial), and total bout time (sec; the sum of all individual cognitive tunneling bout durations within a trial). Gaze behavior metrics were collected during both phases of flight (cruise, landing) and under single flight task and dual task conditions. Therefore, these data were subjected to a 2×2 repeated measures ANOVA (within-subject factors: Task Condition [single, dual], Phase of Flight [cruise, landing]) to determine the impact of cognitive load on gaze behavior. If cognitive load influences gaze behavior during aircraft operation, it is expected to be demonstrated by a reduction in SGE, GTE, an increase in cognitive tunneling and a specific increase in dwell time toward fewer, task-relevant AOIs as cognitive load increases. These findings are expected to be more significant in the landing phase of flight compared to the cruise phase of flight.

Situation awareness (SA) was assessed using a subjective questionnaire. The SART questionnaire (Taylor and Selcon, 1990) is a post-trial self-report questionnaire that uses a 7-point Likert scale (1 = Low; 7 = High) across 10 dimensions of SA. This is collapsed into three larger dimensions of attentional demands, attentional supply and situation understanding. These ratings are then combined to calculate an overall measure of SA.


(5) $$SA=\mathrm{Understanding}-\left(\mathrm{Demand}-\mathrm{Supply}\right)$$

SART scores were post-trial surveys that were completed at the end of a trial and thus could not be partitioned into cruise and landing flight phases. As such, SART scores were subjected to a repeated measures ANOVA (within-group factor: task condition [single, dual]).

All ANOVAs were performed with an alpha level set at 0.05. The Bonferroni *post hoc* correction for multiple comparisons was also applied for all *post hoc* analyses following the repeated measure ANOVAs to determine significant differences between variables. Table 1 provides a summary of all the ANOVAs conducted and their respective independent variables.

**Table 1:** List of dependent measures and their associated ANOVAs and independent variables.

Dependent variable	ANOVA	Independent variables
Flight performance metrics	1×2	Task Condition (single flying, dual task)
Raw auditory performance metrics	1×3	Cognitive Load (single auditory, dual cruise, dual landing)
Dual-task cost auditory scores	1×2	Phase of Flight (cruise, landing)
Gaze behavior metrics	2×2	Task Condition (single flying, dual task) Phase of Flight (cruise, landing)
SART scores	1×2	Task Condition (single flying, dual task)

The listed dependent variables represent the larger clusters of actual dependent variables. Flight performance metrics include landing performance metrics (total completion time, landing error, landing hardness), as well as cruise performance metrics (airspeed and vertical speed mean and variability). Raw auditory performance metrics include response time and response accuracy. Dual task cost auditory scores include auditory response cost and auditory accuracy cost. Gaze behavior metrics include dwell time, fixation duration, SGE, GTE, cognitive tunneling bout number, duration, and total time. SART scores include overall SA, SA supply, SA demand, and SA understanding scores.

## Results

### Flight task performance

Flight performance measures were subjected to a repeated measures ANOVA (within-group factor: task condition [single, dual task]). Cruise aircraft performance results demonstrated no significant differences between the single and dual task for cruise airspeed mean and variability, *F*(1, 20) = 1.594 and 0.091, *p =* 0.224 and 0.767, 𝜂_p_^2^ = 0.087 and 0.005, or cruise vertical speed mean and variability, *F*(1, 20) = 0.613 and 1.407, *p* = 0.444 and 0.252, 𝜂_p_^2^ = 0.035 and 0.076, respectively.

Landing performance results demonstrated no significant differences between the single and dual task for completion time, *F*(1, 20) = 1.308, *p* = 0.266, 𝜂_p_^2^ = 0.061, and landing accuracy, *F*(1, 20) = 0.126, *p* = 0.727, 𝜂_p_^2^ = 0.006. However, landing hardness was significantly higher (i.e., more negative) in the dual task, *F*(1, 20) = 8.562, *p* = 0.008, 𝜂_p_^2^ = 0.300. All means and standard deviations for cruise and landing flight performance measures are reported in Table 2.

**Table 2:** Flight performance values for single and dual task conditions.

Dependent variable	Single	Dual
Completion time (sec)	494.1 (22.2)	491.2 (25.35)
Landing accuracy (°)	0.055 (0.059)	0.054 (0.049)
Landing hardness (fpm)	−95.56 (46.3)**	−117.1 (51.35)**
Cruise airspeed (kts)	104.4 (0.85)	104.6 (0.83)
Cruise airspeed variability	0.50 (0.44)	0.46 (0.37)
Cruise vertical speed (fpm)	−3.77 (15.53)	−3.14 (14.64)
Cruise vertical speed variability	2.21 (1.90)	2.92 (2.16)

Mean (standard deviation) flight performance values are reported for single and dual task conditions. *p ≤ 0.05, **p ≤ 0.01, ***p ≤ 0.001.

### Auditory task performance

Raw auditory performance scores were subjected to a repeated measures ANOVA with a single within-subject factor (cognitive load: single, dual cruise, dual landing). Dual task cost scores were subjected to a repeated measures ANOVA with phase of flight (cruise, landing) as the only within-subject factor.

Auditory oddball task performance results demonstrated a significant main effect of cognitive load for response time and response accuracy, *F*(2, 40) = 30.914 and 23.648, *p* < 0.001 and < 0.001, 𝜂_p_^2^ = 0.607 and 0.542, respectively. Specifically, response time increased as a function of cognitive load (single = 752.17 msec, SD = 393.16; dual cruise = 1079.26 msec, SD = 487.44; dual landing = 1450.71 msec, SD = 574.79). Response accuracy decreased as a function of cognitive load (single = 99.6%, SD = 0.8; dual cruise = 98.3%, SD = 2.9, dual landing = 90.9%, SD = 7.5).

**Figure 3:**
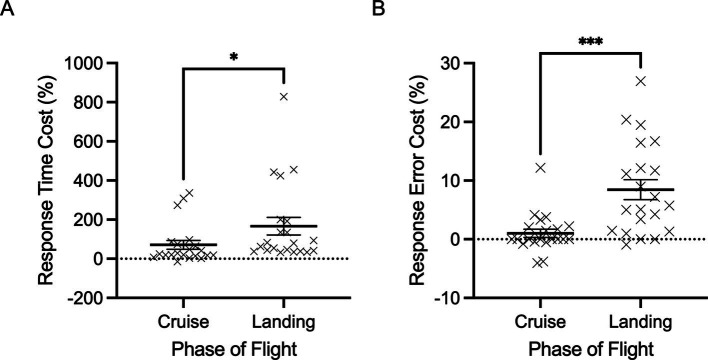
Individual data points and their respective group means for auditory response time cost (%) (A) and response error cost (%) are demonstrated for cruise and landing phases of flight. (B) Error bars represent SEM. *p ≤ 0.05, **p ≤ 0.01, ***p ≤ 0.001.

Figures 3A,B illustrate the dual task costs associated with auditory response time and response accuracy, which demonstrated a main effect of phase of flight, *F*(1,20) = 6.106 and 23.039, *p* = 0.027 and < 0.001, 𝜂_p_^2^ = 0.234 and 0.535, respectively. Specifically, response time cost increased significantly from cruise (mean = 71.2%, SD = 102.7) to landing (mean = 166.3%, SD = 205.3). This is consistent with the response accuracy cost (i.e., this is reflected as the inverse of the actual accuracy difference to express increased ‘cost’ to performance), which is also shown to increase significantly from cruise (mean = 1.3%, SD = 3.1) to landing (mean = 8.7%, SD = 7.6).

### Gaze behavior

**Figure 4:**
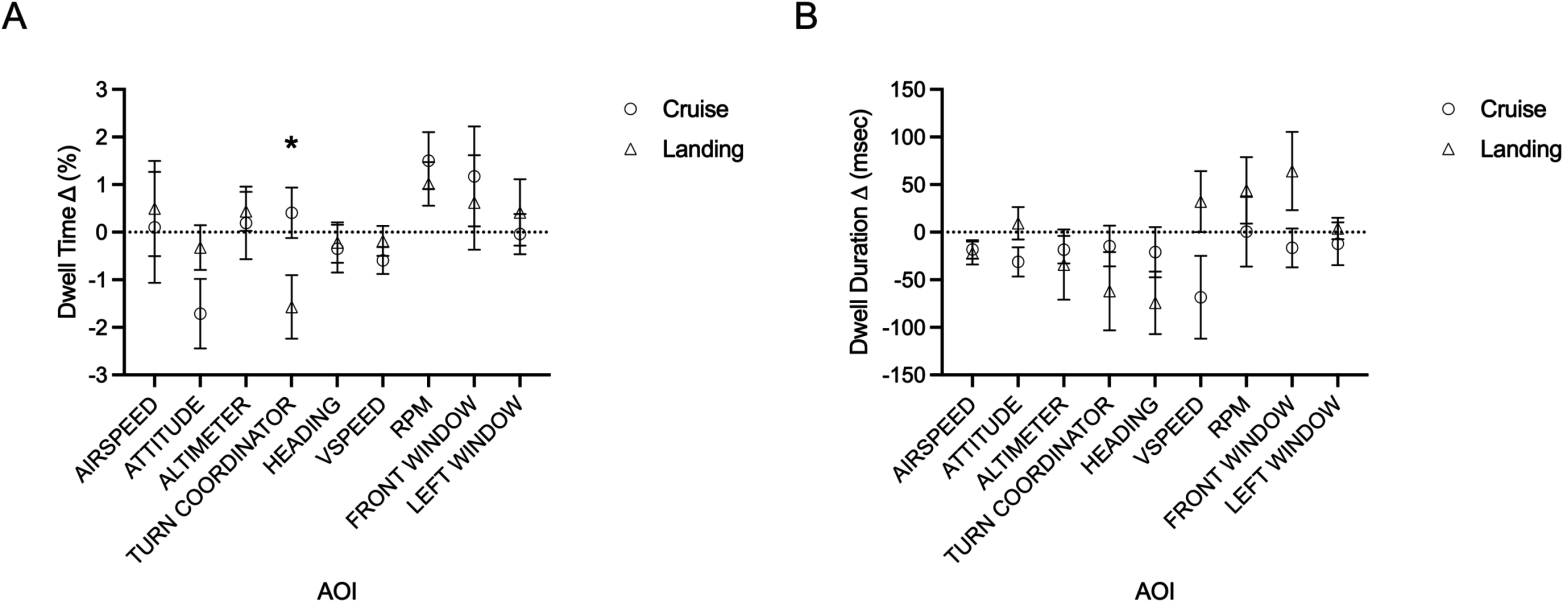
Group means for dwell time difference (%) (A) and dwell duration (msec) difference (B) (*Δ* difference: dual-single) across all areas of interest (AOIs) during cruise (open circle) and landing (open triangle) phases of flight. Error bars represent SEM. Significant interactions between task and flight phase are indicated accordingly *p ≤ 0.05, **p ≤ 0.01, ***p ≤ 0.001.

Gaze metrics were assessed via a 2×2 repeated measures ANOVA (within-group factors: task condition [single, dual task], phase of flight [cruise, landing]). Dwell time (%) demonstrated a main effect of flight phase across several AOIs (Table 3). This included significant increases in dwell time toward airspeed and left window AOIs, as well as significant reductions in dwell time across altimeter, heading, and front window AOIs from the cruise phase to the landing phase of flight. All dwell time means, standard deviations (SD), and statistical test values for the main effect of phase of flight are presented in Table 3. Notably, dwell time toward the power gauge demonstrated a main effect of task, *F*(1,20) = 7.417, *p* = 0.013, 𝜂_p_^2^ = 0.271. Specifically, dwell time was higher during the dual task (mean = 6.67%, SD = 0.73) compared to the single task (mean = 5.41%, SD = 0.49). Dwell time toward the turn coordinator demonstrated an interaction between task and flight phase, *F*(1,20) = 6.689, *p* = 0.018, 𝜂_p_^2^ = 0.251. Post-hoc decomposition of this interaction demonstrated that dwell time toward the turn coordinator was significantly higher for the cruise phase during the flying only (i.e., single) task (mean = 8.58%, SD = 4.34) compared to the dual task (mean = 6.87%, SD = 2.86), while this remained relatively consistent across tasks during the landing flight phase (single: mean = 6.61%, SD = 3.47; dual: mean = 6.29%, SD = 3.01). No other effects of flight phase, task condition, or interactions between the two reached statistical significance (*p* > 0.05). The lack of main effect or interaction involving task condition for dwell time are illustrated in Figure 4A. As such the mean (SD) and group statistics were collapsed across task conditions and reported in Table 3 for each phase of flight.

**Table 3:** Dwell time (%) descriptive and group statistics.

Area of interest (AOI)	Cruise	Landing	F statistic	*P* value	Eta squared
Airspeed	6.77 (5.45)	16.43 (7.06)	77.293	<0.001	0.794
Altimeter	12.72 (6.14)	6.12 (2.47)	44.94	<0.001	0.692
Heading	4.99 (4.39)	2.52 (1.48)	9.547	0.006	0.323
Front window	40.88 (15.31)	35.37 (7.47)	6.241	0.021	0.238
Left window	4.61 (2.38)	16.42 (5.59)	119.21	<0.001	0.856

Mean (standard deviation) dwell time (%) values across significant areas of interest (AOI) are reported across cruise and landing phases of flight, which were collapsed across single and dual task conditions since no main effect of task condition was reported. Statistical test values (F, *p*, 𝜂_p_^2^) for main effects of phase of flight (i.e., cruise, landing) are reported for only significant AOIs. Df (1,20).

Results for average dwell duration are shown in Table 4. There was a main effect of flight phase across several AOIs. This included significant increases in fixation duration across airspeed, turn coordinator, and front window AOIs, along with a significant decrease in fixation duration toward the altimeter AOI. Airspeed also demonstrated a main effect of task, *F*(1,20) = 5.983, *p* = 0.024, 𝜂_p_^2^ = 0.024, which specifically demonstrated a reduction in fixation duration during the dual task (mean = 480.72 msec, SD = 73.42) compared to the flying only condition (mean = 500.67 msec, SD = 87.39). All other AOIs were not significantly modulated by task condition or phase of flight (*p* > 0.05). The lack of main effect or interaction involving task condition for dwell duration are illustrated in Figure 4B. As such the mean (SD) and group statistics were collapsed across task conditions and reported in Table 4 for each phase of flight.

**Table 4:** Average dwell duration (msec) descriptive and group statistics (SEM).

Area of interest (AOI)	Cruise	Landing	F statistic	*p* value	Eta squared
Airspeed	428.88 (85.73)	552.51 (107.18)	24.657	<0.001	0.552
Altimeter	502.67 (101.32)	457.99 (104.67)	4.985	0.037	0.2
Turn coordinator	431.69 (146.78)	496.93 (146.77)	5.88	0.025	0.227
Front window	657.22 (172.90)	888.88 (284.48)	12.572	0.002	0.386

Mean (standard deviation) average dwell duration (msec) values across significant areas of interest (AOI) are reported across cruise and landing phases of flight. Statistical test values (F, *p*, 𝜂_p_^2^) for main effects of phase of flight (i.e., cruise, landing) are reported for only significant AOIs. Df (1,20).

**Figure 5:**
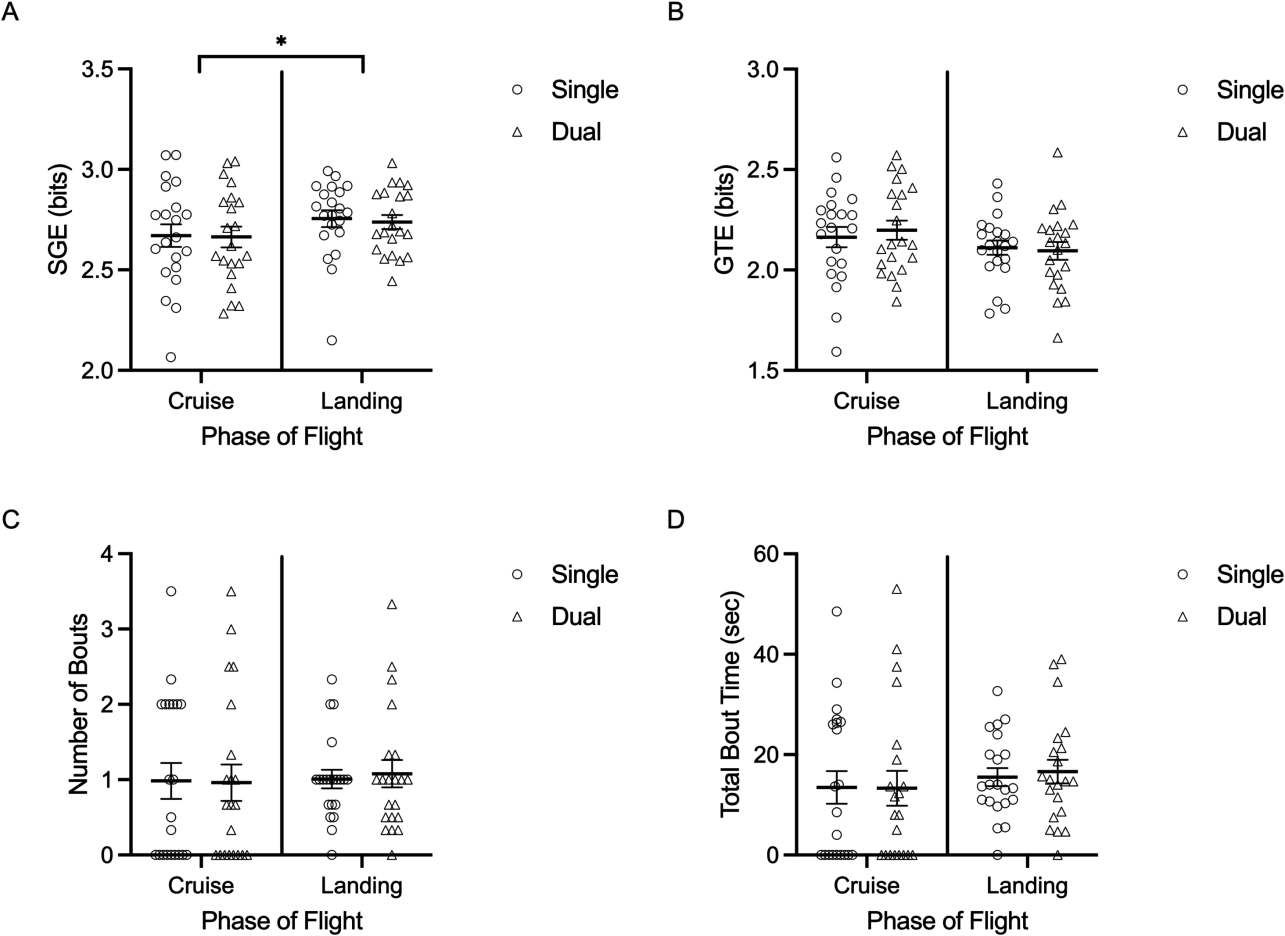
Individual data points and their respective group means for stationary gaze entropy (SGE) (A), gaze transition entropy (GTE) (B), number of cognitive tunneling bouts (C), and total cognitive tunneling bout time (sec) (D) are demonstrated for both task conditions (single, dual) across cruise and landing phases of flight. Error bars represent SEM. *p ≤ 0.05, **p ≤ 0.01, ***p ≤ 0.001.

SGE (bits) showed a main effect of flight phase, *F*(1,20) = 5.311, *p* = 0.0332, 𝜂_p_^2^ = 0.210, demonstrating that fixation dispersion was significantly greater during landing (mean = 2.74 bits, SD = 0.16) compared to the cruise phase (mean = 2.66 bits, SD = 0.24) (Figure 5A). However, SGE and GTE were not significantly impacted by task, and there was no interaction with flight phase (*p* > 0.05). Similarly, there were no significant main effects of task, flight phase or interaction for cognitive tunneling bout metrics (*p* > 0.05) (Figure 5).

### Subjective situation awareness ratings

**Figure 6:**
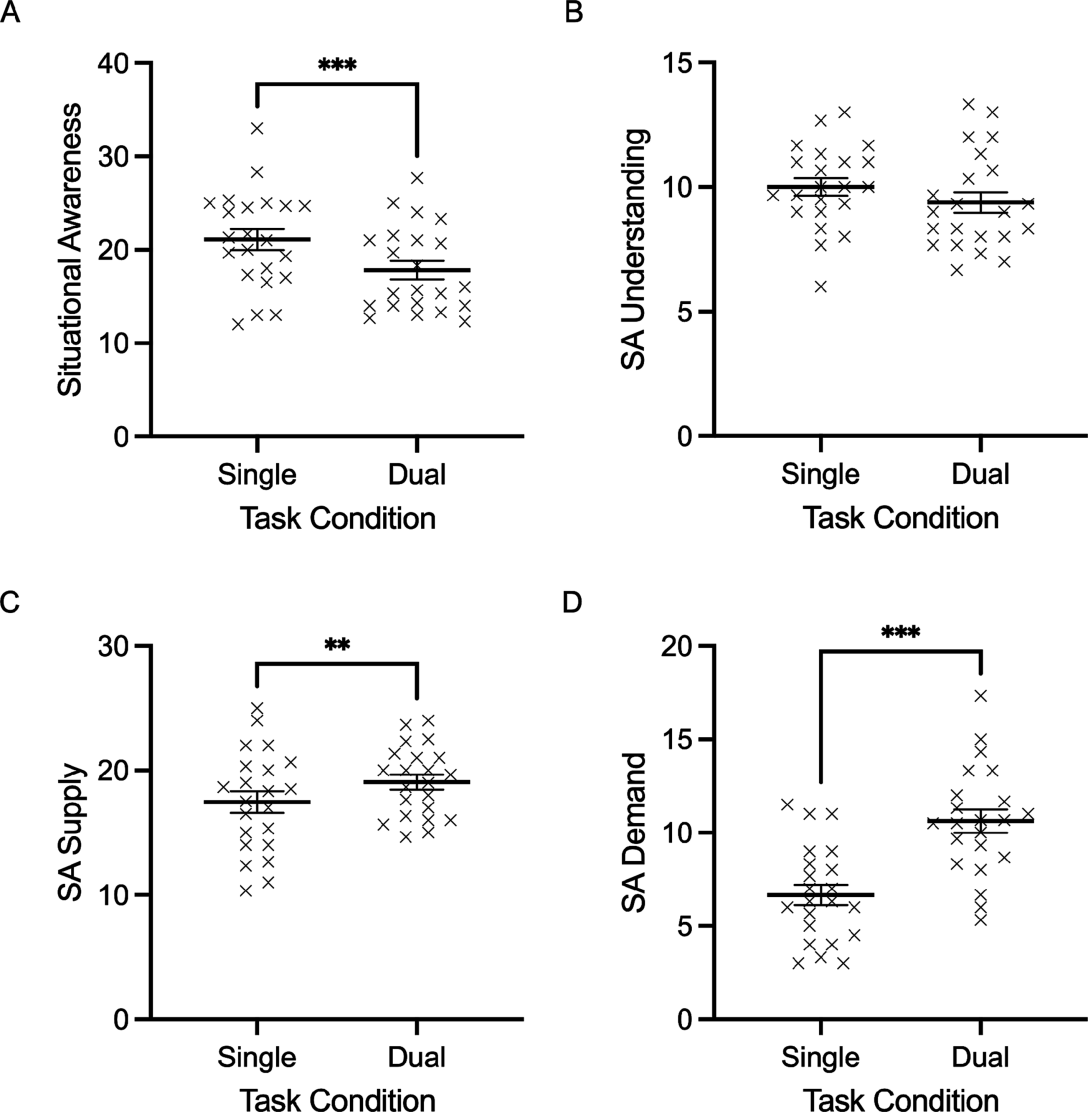
Individual data points and their respective group means for situation awareness (SA) (A), SA understanding (B), SA supply (C), and SA demand are demonstrated for single flying and dual task conditions. (D) Error bars represent SEM. *p ≤ 0.05, **p ≤ 0.01, ***p ≤ 0.001.

SART scores were examined with a repeated measures ANOVA with a single within-subject factor (task condition: single, dual). Participant self-ratings of situation awareness demonstrated a main effect of task, *F*(1,20) = 31.199, *p* < 0.001, 𝜂_p_^2^ = 0.609. Participants reported higher subjective situation awareness in the single flying condition (mean = 21.5, SD = 5.0) than in the dual task (mean = 18.0, SD = 4.6) (Figure 6). The breakdown of situation awareness scoring also demonstrated main effects of task for attentional demand and attentional supply, *F*(1,20) = 49.021 and 7.827, *p <* 0.001 and 0.012, 𝜂_p_^2^ = 0.710 and 0.281, respectively (Figure 6). Specifically, attentional demand demonstrated a significant increase from the single flying condition (mean = 6.30, SD = 3.17) to the dual task (mean = 10.73, SD = 2.95). This was seen in parallel with a significant increase in attentional supply from the single (mean = 17.69, SD = 4.03) to the dual task (mean = 19.28, SD = 2.69). No significant differences were reported for task understanding (*p* = 0.054).

## Discussion

Pilot monitoring behavior is a critical aspect of safe aircraft operation. Understanding where and when pilots allocate their attention are important aspects of information processing. Specifically, gaze analysis could help to understand a pilot’s ability to use task-relevant information in the environment to assess, plan and generate the necessary sequence of actions required at any given moment. The current study examined the effect of a dual task on flight performance and gaze behavior during two phases of flight with differing levels of cognitive load. Participants reported the dual task was more challenging as indicated by the SART questionnaire. Notably, the auditory task revealed that the landing phase imposed a higher cognitive load compared to the cruise phase as demonstrated by reduced accuracy and longer response times to auditory tones. In contrast to our hypotheses, flight performance and most of the gaze behavior indicators were not significantly affected by the dual task suggesting that the type of secondary task used and the way it is introduced plays a role in the impact it has on the primary task. Therefore, this finding suggests that the pilot cohort tested in this study attained a level of proficiency that allowed them to effectively deploy their cognitive resources to complete the flying task across all task manipulations. These findings are discussed in the context of evaluating gaze behavior as a marker of information processing efficiency.

Although SA and auditory task performance confirmed that the dual task increased cognitive load as hypothesized, responding to the auditory tones had very small effects on flight performance and gaze behavior metrics in both the cruise and landing flight phases. The flight performance results confirm that participants followed instructions to make flying the aircraft the primary task but indicate that the secondary task was not challenging enough to impair performance on the primary task, as has been demonstrated in other literature employing a dual-task paradigm (Allsop and Gray, 2014; Babu et al., 2019; Engström et al., 2017; Lutnyk et al., 2023; Yang et al., 2018; Yang et al., 2022). The only significant difference in flight performance was an increase in landing hardness during the dual task. However, the approximately 22 fpm increase in landing hardness is not perceptible, nor did it deem the landings unsafe given that the aircraft in question can handle landings with a landing hardness of 700 fpm.

With respect to the gaze behavior findings, a few significant differences were reported between single and dual tasks. First, average dwell durations were shown to decrease significantly (~20 msec) during the dual task, specifically for the airspeed gauge. According to previous work, this finding suggests that participants required less time to process airspeed AOI information in this condition (Ayala et al., 2022; Brams et al., 2018; Glaholt, 2014). This may be taken as evidence suggesting a change in gaze strategies to optimize the scanning of gauges (i.e., completing quick checks for task relevant AOIs only to ensure alignment with expected values) while allocating attentional resources to the secondary auditory task. However, this was only shown across a single AOI, which does not provide sufficient evidence that information processing became more efficient in general during the dual task condition. Additionally, the total time taken to complete these scenarios was not different between single and dual task conditions, so it is not a matter of reducing dwell duration time to match the reduced time taken to complete the task. Second, dwell time was significantly higher toward the power gauge during the dual task condition. This increase in power dwell time was reported alongside an interaction effect for the turn coordinator gauge. Specifically, it was demonstrated that dwell time was highest for the turn coordinator during the single task cruise flight phase. Therefore, it seems as though the dual task condition caused a shifting of attention from the turn coordinator to the power gauge. This shift in gaze, however, is only shown to be true during cruise, which is an interesting finding considering that the turn coordinator is primarily used as a visual reference to gauge the rate of turn (or lack of turn during cruise) whereas the power gauge is primarily used to get a sense of engine power and fuel efficiency (Federal Aviation Administration, 2021). As a result of no significant reductions in dwell time shown across any other AOIs because of task condition, it is likely that it came from a number of AOIs, which collectively resulted in an increase in dwell time toward the power gauge during approach and landing. In any case, since these changes in dwell time were quite small (~1–1.5%) and they were not accompanied by a significant reduction in fixation distribution (SGE), there is no evidence to suggest that these changes were associated with a narrowing of attention toward fewer task critical AOIs during states of high cognitive load. Taken together, the reported changes in gaze behavior may reflect minor changes in information processing that helped maintain flight performance and scanning of task critical AOIs across single and dual task conditions during cruise. However, the significance of these changes in gaze behavior and their impact on task performance is not clear and require further research.

In contrast to the traditional gaze metrics, the more dynamic measures of gaze dispersion (SGE), gaze sequence complexity (GTE), and cognitive tunneling (i.e., poor gaze monitoring characterized by a lack of instrument scanning and long dwells outside the cockpit) demonstrated no significant differences between single and dual tasks, nor did they show any interaction with phase of flight to suggest that the task condition had a significantly larger impact on gaze during the approach and landing phase of flight. Instead, the distribution of fixations (SGE) demonstrated an increase in the allocation of attention across a wider range of AOIs during approach and landing compared to cruise, which was associated with a number of changes in traditional measures of gaze behavior (i.e., dwell time and fixation duration). Given that these changes were not associated with the increase in cognitive load from the task condition manipulation or an interaction with phase of flight, we propose that the findings reflect the changes in task objectives and sub-goals between cruise and landing. These findings are in stark contrast to previous work employing a similar set of gaze metrics, which found that increases in task difficulty (i.e., significant crosswinds) (Ayala et al., 2023; Ayala et al., 2024) and cognitive load (i.e., dual task paradigms) (Engström et al., 2017; Kanaan and Moacdieh, 2022; Lu et al., 2020; Yang et al., 2018; Yang et al., 2022) resulted in a narrowing of attention during high difficulty/cognitive load task conditions via reductions in SGE, GTE, saccade amplitudes, visual scanning speed (scan path length per second), along with increases in the percentage of time looking at the center of the external environment (i.e., center of the road or the front window) and cognitive tunneling behavior. A key distinguishing feature between previous dual task work and the current work is the type of secondary task employed. Unlike previous studies that used tasks that are not typically practiced or encountered often in traditional domain-specific routines (i.e., mental arithmetic tasks or the n-back task) (Crosby and Parkinson, 1979; Engström et al., 2017; Kanaan and Moacdieh, 2022; Lu et al., 2020; Yang et al., 2018; Yang et al., 2022), the current auditory task closely resembled the aspect of responding to aircraft specific ATC calls while ignoring ATC calls to other aircraft. It is important to note that the actual ATC calls and verbal responses were replaced with target and non-target tones that required a simple button press, mimicking aspects of auditory perception and identification, without high working memory demands or speech production demands. Accordingly, the lack of verbal responses and memory of relevant call signs (i.e., Whiskey-Alpha-Tango-52) might have resulted in the addition of a secondary task without necessarily increasing the demands on working memory that might have been necessary to see performance deficits in the primary task (Bhojwani et al., 2022; Engström et al., 2017; Lavie, 2005). This finding was similar to precious work done using a dual task driving paradigm (Cao and Liu, 2013), where researchers found that a speech comprehension task alone had minimal impact on driving performance. Evidently, the type of secondary task employed in dual task paradigms affects its impact on primary task performance and gaze measures. This may be accounted for by Baddley’s model of working memory (1986), which suggests that specialized subsystems exist that represent particular types of information. For instance, verbal (i.e., phonological loop) and visuospatial (i.e., visuospatial sketchpad) types of sensory information are structurally independent of one another, and the integrity of information being represented in one domain (i.e., visuospatial demands of flying and aircraft) is not at risk of interference effects from information that may be received and maintained through another domain (i.e., secondary auditory response task). Therefore, we propose that the cognitive load manipulation was not sufficiently challenging to warrant any significant deviations from pilot scan patters between the cognitive load manipulations.

The Cognitive Control Hypothesis (Engström et al., 2017) provides support for an alternative explanation for the lack of performance and gaze findings reported in the current work. Namely, the apparent inconsistencies demonstrated may be reconciled if we were to consider the flight scenario employed to be a task associated with efficient performance and not controlled performance. Since the recruitment pool consisted of pilots who all obtained at least their PPL - thus implying a certain level of proficiency with flying a single engine aircraft-, perhaps their experience with flight circuits (including cruise and landing phases of flight, which are normally accompanied by numerous auditory-verbal ATC calls) may be characterized as well-practiced maneuvers that are refined over the course of training. Thus, it is not a stretch to suggest that task performance is supported during challenging scenarios by improved neural efficiency among low-time pilots who obtain their PPL/CPL certifications. This may fall along a gradient of improved processing and performance efficiencies that are associated with practice-dependent neuroplastic changes. These changes may not necessarily reflect diminished neural involvement in the performed actions, but rather reflect the development of neural connections that facilitate performance speed and accuracy while reducing the cognitive demands of skilled performance (Immink et al., 2020). Thus, highly experienced pilots would be more efficient than those examined in the current work. Since the Cognitive Control Hypothesis states that manipulations to cognitive load selectively impairs controlled performance, the current study likely demonstrates a case wherein increasing cognitive load through a secondary task left the primary flying subtasks and pilot gaze behavior mostly unaffected. Indeed, work by Nibbeling et al. (2012) demonstrated similar findings when examining the effects of anxiety and a dual task paradigm on a dart throwing task across novice and expert players. Specifically, primary task performance, perceived effort and gaze behavior were negatively impacted by anxiety and cognitive load in novice players whereas expert players experienced little-to-no changes in performance and perceived effort. Although, expert players also demonstrated negative gaze behavior changes, it was less detrimental to information processing compared to novice players. This finding was taken to evince that expert players developed a level of information processing and performance efficiency that spared the use of cognitive resources, which were then used to support task performance when cognitive load and anxiety increased. Notably, research examining the extent to which improved processing and performance efficiency impacts gaze behavior during states of high cognitive load is sparse and requires further investigation.

### Limitations and future directions

This work was constrained by two main methodological limitations. First, the method through which the auditory tone task was implemented was done because it was easy to conduct and control. However, it did not fully represent the verbal perception and speech production aspects of ATC communication that likely added to the working memory load that participants would have had to manage. Although alternative accounts have been suggested, this might have played a role in why the dual task paradigm employed was not sufficiently challenging for the recruited pilot group. Naturally, this produced additional questions for future research about its utility in providing an objective measure of pilot monitoring behavior and flight proficiency. Specifically, experienced pilots who have reached a level of information processing and performance efficiency with the evaluated flight maneuvers will have additional cognitive control resources to allocate toward secondary cognitively demanding tasks. In contrast, less experienced pilots who are still performing the same flight maneuvers at a controlled level of performance will have fewer cognitive control resources to allocate toward a secondary cognitively demanding task and will likely show larger task related and information processing impairments. The extent to which this will become evident in pilot monitoring behaviors via gaze analyses requires further exploration. Another direction for future research is to examine the effect of a dual-task paradigm using an auditory secondary task that more realistically simulates ATC verbal communication. To that point, this work provides a necessary empirical stepping stone from which auditory ATC call outs can be used in place of auditory tones to not only increase cognitive load sufficiently, but also to increase fidelity (i.e., mimicking a busy airspace with frequent ATC calls being conducted to several aircraft). Furthermore, a more significant limitation associated with the current work is connected to the way in which auditory response time data were collected. The auditory tones were created and recorded using an external device VPixx, while the hardware used to collect the responses was implemented in the ALSIM simulator. Therefore, the start and stop of trials across these various streams of stimulus presentation and data collection were tightly coordinated between two researchers to ensure button presses (to start and stop trial) were occurring at the same time. Nevertheless, human variability exists in simple button presses (Stergiou and Decker, 2011) and the synchronization of the auditory tones with responses was likely not perfectly synchronized. Future work should incorporate stimulus presentation and response recording in the simulator environment to get a more accurate recording of auditory tone responses. Despite this limitation, it is important to note that the auditory response accuracy data reported in the current study echoes previous work in demonstrating the presence a dual task cost that is further exaggerated under more demanding task conditions. This is a finding that was not contaminated by the synchronization limitation and thus provides support in the auditory task findings reported here (Allsop and Gray, 2014; Allsop et al., 2017; Crosby and Parkinson, 1979; Lutnyk et al., 2023; Yang et al., 2022).

## Conclusion

The present paper investigated the utility of gaze behavior in assessing changes in cognitive load within a simulated flying task using a dual task paradigm. Auditory task performance demonstrated that increases in cognitive load differentially impacted the cruise and landing phases of flight. Specifically, dual task cost in response time and response errors was higher in the approach and landing phase of flight compared to cruise. The increase in task demands across flight phases was associated with an increase in average dwell duration across AOIs that were crucial to the phase specific sub-tasks. However, since most gaze behavior metrics and flight performance remained unchanged when the dual task was introduced it is likely that the recruited pilot group was not sufficiently challenged. These findings provide guidance for future research in this area with regard to the types of tasks that should be used for creating measurable levels of cognitive load at the low-time pilot level, while also shedding light on the efficacy of utilizing dual task paradigms to assess pilot information processing and flight proficiency.

## Data Availability

The raw data supporting the conclusions of this article will be made available by the authors, without undue reservation.
